# Spectroscopic investigations of a semi-synthetic [FeFe] hydrogenase with propane di-selenol as bridging ligand in the binuclear subsite: comparison to the wild type and propane di-thiol variants

**DOI:** 10.1007/s00775-018-1558-4

**Published:** 2018-04-07

**Authors:** C. Sommer, S. Rumpel, S. Roy, C. Farès, V. Artero, M. Fontecave, E. Reijerse, W. Lubitz

**Affiliations:** 1 Max-Planck-Institut für Chemische Energiekonversion, Stiftstrasse 34-36, 45470 Mülheim an der Ruhr, Germany; 2 Laboratoire de Chimie et Biologie des Métaux, Université Grenoble Alpes, CEA/BIG, CNRS, 17 rue des martyrs, 38000 Grenoble, France; 3 Max-Planck-Institut für Kohlenforschung, Kaiser-Wilhelm Platz 1, 45470 Mülheim an der Ruhr, Germany; 4 Laboratoire de Chimie des Processus Biologiques, Collège de France, Université Pierre et Marie Curie, CNRS, UMR 8229, PSL Research University, 11 place Marcelin Berthelot, 75005 Paris, France

**Keywords:** [FeFe] Hydrogenase, Chalcogenic substitution, Nuclear magnetic resonance, Electron paramagnetic resonance, FTIR spectroelectrochemistry

## Abstract

[FeFe] Hydrogenases catalyze the reversible conversion of H_2_ into electrons and protons. Their catalytic site, the H-cluster, contains a generic [4Fe–4S]_H_ cluster coupled to a [2Fe]_H_ subsite [Fe_2_(ADT)(CO)_3_(CN)_2_]^2−^, ADT = µ(SCH_2_)_2_NH. Heterologously expressed [FeFe] hydrogenases (apo-hydrogenase) lack the [2Fe]_H_ unit, but this can be incorporated through artificial maturation with a synthetic precursor [Fe_2_(ADT)(CO)_4_(CN)_2_]^2−^. Maturation with a [2Fe] complex in which the essential ADT amine moiety has been replaced by CH_2_ (PDT = propane-dithiolate) results in a low activity enzyme with structural and spectroscopic properties similar to those of the native enzyme, but with simplified redox behavior. Here, we study the effect of sulfur-to-selenium (S-to-Se) substitution in the bridging PDT ligand incorporated in the [FeFe] hydrogenase HydA1 from *Chlamydomonas reinhardtii* using magnetic resonance (EPR, NMR), FTIR and spectroelectrochemistry. The resulting HydA1-PDSe enzyme shows the same redox behavior as the parent HydA1-PDT. In addition, a state is observed in which extraneous CO is bound to the open coordination site of the [2Fe]_H_ unit. This state was previously observed only in the native enzyme HydA1-ADT and not in HydA1-PDT. The spectroscopic features and redox behavior of HydA1-PDSe, resulting from maturation with [Fe_2_(PDSe)(CO)_4_(CN)_2_]^2−^, are discussed in terms of spin and charge density shifts and provide interesting insight into the electronic structure of the H-cluster. We also studied the effect of S-to-Se substitution in the [4Fe–4S] subcluster. The reduced form of HydA1 containing only the [4Fe–4Se]_H_ cluster shows a characteristic S = 7/2 spin state which converts back into the S = 1/2 spin state upon maturation with a [2Fe]–PDT/ADT complex.

**Electronic supplementary material:**

The online version of this article (10.1007/s00775-018-1558-4) contains supplementary material, which is available to authorized users.

## Introduction

**Fig. 1:**
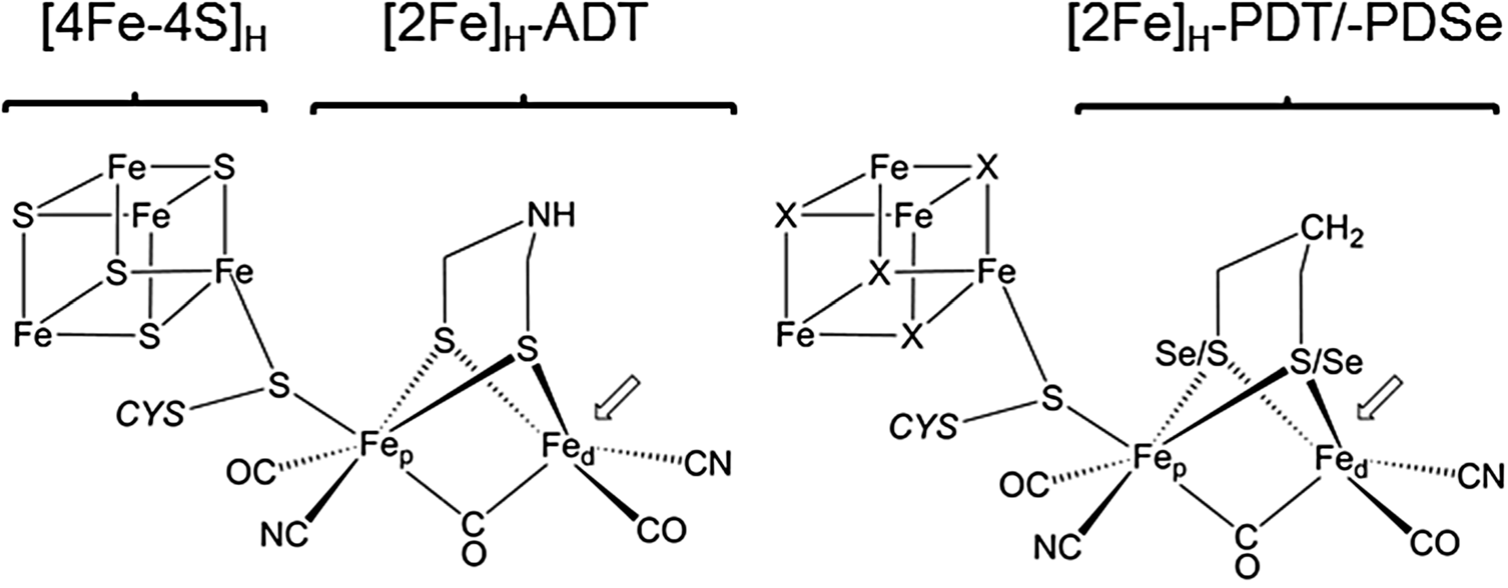
The native active site of [FeFe] hydrogenase and applied modifications. The iron atoms are labeled as proximal (Fe_p_) and distal (Fe_d_) with respect to their position to the [4Fe–4S]_H_ cluster. Left: native H-cluster that consists of the [4Fe–4S]_H_ cluster and [2Fe]_H_-ADT subsite. Right: modified H-cluster with [2Fe]_H_-PDT or [2Fe]_H_-PDSe. Additionally bridging sulfides in the cubane cluster (here marked with X) can be exchanged to Se

The reversible heterolytic splitting of hydrogen into protons and electrons is one of the most fundamental reactions in chemistry. In nature, hydrogen is part of the energy metabolism of several single cellular organisms which are spread over all three domains of life [1, 2]. [FeFe] Hydrogenases catalyze the conversion of protons and electrons into hydrogen in a very efficient way with turnover frequencies over 10,000 H_2_/s [3, 4]. The active site in these enzymes, the so-called H-cluster, consists of a generic [4Fe–4S]_H_ cluster linked to a binuclear iron complex [2Fe]_H_ carrying 3 CO and 2 CN^−^ ligands as well as a bridging aza-propane-dithiolate (ADT) ligand (see Fig. 1) that serves as proton relay of the protein’s proton transport pathway [5]. Often, additional [4Fe–4S] clusters are present that form an electron transport chain connecting the H-cluster with the protein surface where redox partners of the enzyme can bind.

The small hydrogenase from *Chlamydomonas reinhardtii* HydA1 is used as prototype for [FeFe] hydrogenases, since it contains only the H-cluster and can be overexpressed in *Escherichia coli* [6] with high yields [7]. However, since the host organism lacks the maturation factors that built the [2Fe]_H_ subsite, the resulting enzyme (called apo-HydA1) only contains the [4Fe–4S]_H_ cluster and is inactive in hydrogen conversion. Through artificial maturation using a synthetic precursor of the [2Fe]_H_ subsite, the active enzyme can be obtained [8, 9]. This process can be conveniently followed using FTIR spectroscopy, since the CO and CN^−^ stretches of the H-cluster are found in a frequency range that does not overlap with the strong protein amide bands [10]. The use of synthetic precursors of the [2Fe]_H_ subsite opens the possibility of introducing modified complexes into the enzyme and label these with different nuclear isotopes [11–13]. In addition, the generic [4Fe–4S]_H_ cluster can be modified through classical reconstitution [7, 14].

Native HydA1 shows a variety of redox states. The [2Fe]_H_ site can be reduced [Fe_p_(I) Fe_d_(I)] or oxidized [Fe_p_(I) Fe_d_(II)]. Likewise, the [4Fe–4S]_H_ subcluster can be present in reduced (1 +) or oxidized (2 +) forms. Additionally, the bridgehead amino group can be protonated (NH_2_^+^) or unprotonated (NH) [15]. It is assumed that, for the doubly reduced state of the H-cluster, transfer of an NH_2_^+^ proton to the distal iron Fe_d_ affords a terminal hydride that upon reprotonation forms H_2_ [16–18].

The enzyme is inhibited by external CO, which binds to the open coordination site at Fe_d_. This additional donor ligand has a strong effect on the electronic structure of the H-cluster as observed by EPR and FTIR [19]. Furthermore, it has been shown that the redox and catalytic behavior of the active enzyme in various organisms is sensitive to pH [15] and the presence of accessory [4Fe–4S] clusters [20]. To affect and possibly improve the activity and oxygen resistance of [FeFe] hydrogenases, the bridging ADT ligand in the [2Fe]_H_ subsite has been extensively modified through artificial maturation with appropriate synthetic precursors [21, 22]. However, these attempts did not lead to improved enzymatic properties (activity, O_2_ resistance) but did provide useful insight into the structure/function relations of the H-cluster. By substituting the bridgehead amino group to a methylene group, using the [2Fe]-PDT precursor, the [2Fe]_H_ subsite is locked into the mixed valence [Fe_p_(I)Fe_d_(II)] configuration reducing the number of accessible redox states in HydA1-PDT to two: H_ox_ and H_red_. These species are represented as $\left[ { 4 {\text{Fe}} - 4 {\text{S}}} \right]_{\text{H}}^{{ 2 { + }}}$–[Fe_p_(I)Fe_d_(II)] and $\left[ { 4 {\text{Fe}} - 4 {\text{S}}} \right]_{\text{H}}^{ + }$–[Fe_p_(I)Fe_d_(II)], respectively [23]. The spectroscopic signatures (FTIR/EPR) of these states are virtually identical to the corresponding states in the native enzyme, suggesting a very similar electronic and geometric structure. Interestingly, despite the structural similarities with the native enzyme [24], in HydA1-PDT, binding of extrinsic CO to the open coordination site has not been observed.

Inspired by selenium’s role in oxidative protection in [NiFeSe] hydrogenases [25], variants of the H-cluster were recently reported in which the sulfurs in both [4Fe–4S]_H_ and [2Fe]_H_ subclusters were changed to selenium. The atomic mass of Se is more than twice that of sulfur. It has a ≈ 15% increased atomic radius and forms more polarized bonds based on its stronger metallic character compared to sulfur. It has been shown that [4Fe–4Se]_H_ reconstituted HydA1-ADT does not show any decrease in activity nor does it change the vibrational characteristics of the CO and CN^−^ ligands [7]. A S-to-Se substitution of both thiol groups in the [2Fe]_H_ subsite generates an enzyme which shows similar activity but is less stable under laboratory conditions [22].

To study the effect of S-to-Se substitutions on the electronic structure of the H-cluster, we turned to the more stable HydA1-PDSe enzyme, produced via maturation of apo-HydA1 with the [2Fe]-PDSe precursor. HydA1-PDSe, in analogy to HydA1-PDT, shows a stable H_ox_ and a singly reduced H_red_ state. However, in contrast to HydA1-PDT, HydA1-PDSe reacts with CO and forms a H_ox_–CO state. Since the structural properties of HydA1-PDSe are expected to be very similar to those of the native enzyme as is the case for the HydA1-PDT variant, HydA1-PDSe was used to study the effect of S-to-Se exchange on the electronic structure of the H-cluster, using FTIR spectroelectrochemistry, EPR and NMR spectroscopy. ^1^H NMR spectroscopy under ambient conditions (liquid solution) has recently been introduced as a technique to study the hydrogenase active site [26]. For these enzymes ^1^H NMR is very useful, since the active site with its reactants as well as the surrounding protein can be studied simultaneously with atomic (nuclear) resolution under near physiological conditions. NMR has frequently been used as a tool to study paramagnetic iron–sulfur proteins [27, 28] to resolve the electron distribution and the magnetic couplings; for unknown systems it can help to identify the cluster type [29].

We have also studied S-to-Se substitution in the [4Fe–4S]_H_ subcluster using the unmaturated apoenzyme. This substitution often leads to high spin multiplicity in the reduced state of the cluster and is well documented for ferredoxins [30–33].

## Materials and methods

### Synthesis of active site mimics

All reactions were carried out under an inert atmosphere of argon using standard Schlenk and vacuum-line techniques. Solvents were freshly distilled under argon using appropriate drying agents and the distilled solvents were degassed by three freeze–pump–thaw cycles. FTIR spectra of the complexes were recorded on a Perkin Elmer Spectrum-100 spectrometer via a thin film solution using a stainless steel sealed liquid spectrophotometer cell with CaF_2_ windows. [Fe_2_(µ(SeCH_2_)_2_CH_2_)(CO)_6_] was synthesized according to literature procedure [34].

### (Et_4_N)_2_[Fe_2_(µ(SeCH_2_)_2_CH_2_) (CO)_4_(CN)_2_]

To a solution of Fe_2_[(µ(SeCH_2_)_2_CH_2_)(CO)_6_] (0.146 g, 0.3 mmol) in acetonitrile (15 mL), tetraethylammonium cyanide (0.105 g, 0.62 mmol) was added under positive argon flow. After stirring at room temperature for 3 h, the reaction mixture was cannula filtered to a Schlenk flask and the red solution was concentrated to approximately 7 mL. This solution was layered with diethyl ether (20 mL) and cooled to 253 K to yield (Et_4_N)_2_[Fe_2_(µ(SeCH_2_)_2_CH_2_) (CO)_4_(CN)_2_] as a dark red solid. IR (CH_3_CN, cm^−1^): 2075 (CN^−^), 1955, 1918, 1879 (CO).

### (Et_4_N)_2_[Fe_2_(µ(SeCH_2_)_2_CH_2_) (CO)_4_(C^15^N)_2_]

A solution of KC^15^N (0.016 g, 0.24 mmol) in methanol (5 mL) was added dropwise via a cannula to a solution of [Fe_2_(µ(SeCH_2_)_2_CH_2_)(CO)_6_] (0.045 g, 0.1 mmol) in acetonitrile (5 mL). After stirring the reaction mixture for 30 min at room temperature, a solution of [Et_4_N]Br (0.05 g, 0.24 mmol) in acetonitrile (4 mL) was added, and the dark red solution was stirred for 3 h. The solvent was removed under reduced pressure to yield a dark red oily solid. This residue was redissolved in acetonitrile (5 mL) and filtered via cannula to give a dark red filtrate. This solution was layered with diethyl ether (15 mL) and cooled to 253 K to produce (Et_4_N)_2_[Fe_2_(µ(SeCH_2_)_2_CH_2_)(CO)_4_(C^15^N)_2_] as a dark red solid (0.028 g, 25%). IR (CH_3_CN, cm^−1^): 2044 (C^15^N^−^), 1956, 1917, 1879 (CO).

### Protein purification and maturation

Apo**-**HydA1 protein expression and maturation are based on a slightly modified previously published protocol [8, 35]. The pH was adjusted prior to induction of the protein expression and the purification was performed without any dithionite. As selection antibiotic, 30 mg/L kanamycin was used. For maturation the apoprotein was diluted to 350 µM in 0.1 M Tris/HCl, 0.15 M NaCl, pH 8.0, and a threefold excess of [2Fe]-PDSe/-PDT dissolved in DMSO was added and incubated for 1 h. For maturation of [4Fe–4Se]_H_ apo-HydA1 with [2Fe]-PDT/-PDSe, a temperature of 310–311 K and an incubation time of 2–3 h was used. Unbound complexes were removed by a desalting column (PD-10, GE Healthcare) and the maturated proteins were concentrated (Merck Millipore, Amicon Ultra-15, 30 kDa).

### Substitution of [4Fe–4S]_H_ with [4Fe–4Se]_H_ through reconstitution

All steps for cluster reconstitution were performed anaerobically. [4Fe–4S]_H_ apo-HydA1 was unfolded with 6 M guanidinium chloride buffer (0.1 M Tris/HCl, 20 mM disodium EDTA, pH 7.5) to extract the bound cubane cluster. To remove the chaotropic agent, the sample was desalted three times over a PD-10 column (GE Healthcare). While removing the chaotropic agent, apo-HydA1 refolds in the used 0.1 M Tris/HCl, 0.15 M NaCl pH 8.0 buffer. The protein was prepared for the new cluster assembly by reduction with 5 mM dithiothreitol. After reduction, a 12- to 16-fold excess of FeCl_3_ was added followed after 10 min by the same excess of reduced NaSe_2_ into the continuously stirred solution. After incubation for 90 min at room temperature, the solution was dark brown and the reconstitution was stopped. After two consecutive desalting steps, the [4Fe–4Se]_H_ apo-HydA1 was concentrated and used for further applications.

### H_2_ oxidation assay

The hydrogen oxidation assay was performed based on methyl viologen (MV) reduction under strictly anaerobic conditions as previously described [35]. The photometric assay (578 nm) was performed with 100 µg HydA1-PDT/-PDSe in a reaction volume of 1 mL with hydrogen saturated 100 mM K_*x*_H_*y*_PO_4_, 10 mM MV, pH 6.8 buffer. The slope was determined and the activities were calculated based on an extinction coefficient for the MV radical of 9780 M^−1^ cm^−1^.

### H_2_ production

Hydrogen production was examined analogous to Winkler et al. [36] using gas chromatography. In a 2 mL gas-tight vial, 400 µL reaction volume containing 10 µg HydA1-PDT/-PDSe, 300 mM K_*x*_H_*y*_PO_4_ pH 6.8, 10 mM MV and 100 mM sodium dithionite (NaDT) was flushed with argon for 5 min. The samples were incubated at 310 K for 20 min. A gas chromatogram of 300 µL headspace was recorded at 313 K with an RT-MSieve 5 Å column. Given are the mean values with standard deviations calculated from three measurements per sample in triplicate.

### FTIR spectroscopy and FTIR spectroelectrochemistry

Transmission FTIR spectra were obtained using a Vertex 80v FTIR spectrometer from Bruker Optics with an N_2_ cooled mercury cadmium telluride (MCT) detector. All sample preparations were performed under strict anaerobic conditions. Samples were immobilized between CaF_2_ windows and measured in a continuously purged sample chamber. Spectra were recorded with 20 kHz velocity in double-sided forward backward mode with phase resolution of 16, zero filling factor of 2 and Blackman–Harris three-term apodization. Final data processing was performed using home-written scripts in the Matlab^®^ programming environment. FTIR spectroelectrochemistry was carried out as previously described, but without use of redox mediators [15]. Spectra were recorded on a Bruker IFS 66v/s spectrometer with N_2_ cooled MCT detector with an aperture of 2.5–3 mm and thermostated sample (278 K). An equilibration time of 40–60 min was used between the two applied potentials (Autolab PGSTAT101; NOVA software).

### Sample preparation and NMR spectroscopy

For proton NMR spectroscopy, samples were maturated as described and then three times rebuffered to a low salt D_2_O buffer (25 mM K_*x*_H_*y*_PO_4_, pD 6.8) and subsequently concentrated up to 2.5 mM. Finally, the required redox states were titrated with NaDT or thionine acetate and monitored by FTIR spectroscopy. All NMR spectra were acquired on a Bruker AVANCE 600 spectrometer equipped with a cryogenic TCI probehead. The 1D spectra were recorded with 2048 scans, a relaxation delay of 0.2 s and a spectral width of 200 ppm. Spectra were processed and analyzed using Topspin 2.1 and Mnova 10.0.2.

### EPR spectroscopy

X-band CW-EPR spectra were recorded on a Bruker Elexsys 500 EPR spectrometer equipped with a standard TE102 rectangular resonator and an Oxford ESR900 helium flow cryostat.

Pulse echo detected EPR spectra were obtained using a Bruker Elexsys 580 X-band pulsed EPR spectrometer. Samples were accommodated in a Bruker MD5 dielectric resonator inserted into an Oxford CF935 helium flow cryostat. Q-band Echo detected experiments were conducted on a Bruker Elexsys 580 Q-band pulsed EPR spectrometer using a homebuilt Q-band resonator [37]. Low temperatures were reached using a closed cycle Helium cryostat from Cryogenic Ltd [38].

## Results and discussion

For all artificial enzymes, no catalytic activity for hydrogen production or oxidation was observed (see fig. S1). This is surprising since HydA1-ADSe was reported to be fully active [22]. Therefore, it was anticipated that HydA1-PDSe would at least show an activity similar to that of HydA1-PDT. However, since for the PDT variant of [FeFe] hydrogenases the amine function is lacking, another proton shuttle should be in operation to explain its residual activity. It is speculated that the coordinating bridging thiols, similar to the situation in [NiFe] hydrogenases [1], can temporarily store the proton associated with the hydride. For the PDSe variant, this mechanism seems to fail, probably because of the substantial increase in Fe–Se/S bond length and/or the unfavorable acid/base properties of the selenol moieties.

### The H_ox_ and H_red_ states

The maturation with [2Fe]-PDSe and the charge distribution of the two main states H_ox_ and H_red_ from HydA1-PDT and -PDSe were studied with FTIR spectroscopy using the CO and CN^−^ vibrations as probes.

#### FTIR spectroscopy

The incorporation of the [2Fe]-PDSe precursor into apo-HydA1 was confirmed by FTIR spectroscopy showing narrow CO and CN^−^ bands as compared to those of the free synthetic precursor in solution (see fig. S2A).

**Fig. 2:**
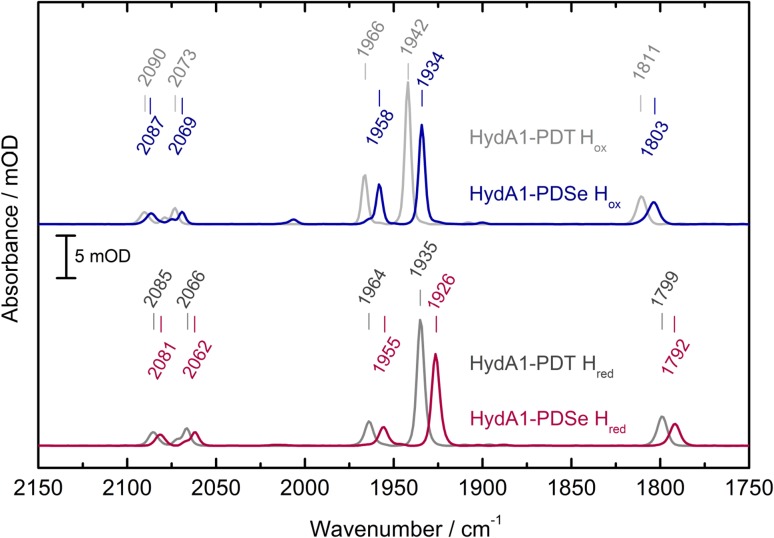
Comparison of the H_ox_ (gray/blue) and H_red_ (dark gray/red) states of [4Fe–4S]_H_ HydA1-PDT/-PDSe in FTIR spectroscopy. The smaller contribution at 2005 cm^−1^ in the spectrum of HydA1-PDSe H_ox_ originates from the oxidized CO inhibited state (see Fig. 5). Spectra are taken at 288 K with a spectral resolution of 2 cm^−1^

As shown in Fig. 2, the signal pattern for CN^−^ and CO bands of the H_ox_ state of HydA1-PDSe is redshifted by 3–8 cm^−1^ compared to HydA1-PDT, which is in good agreement with the redshifts found for the [2Fe]–ADSe complex incorporated into HydA1 from *Clostridium pasteurianum* (*Cp*) by Kertess et al. [22] compared to HydA1-ADT. This can be explained by the donation from the Se lone pair to the Fe σ*-orbital that enhances π-back-donation from Fe to CO/CN^−^ and weakens the CO/CN^−^ internal ligand bond strength.

Reduction with 5 mM NaDT results in HydA1-PDSe H_red_ with small redshifts in the FTIR spectrum compared to the oxidized state (see Table 1) similar to the ones observed for HydA1-PDT upon reduction (see Fig. 2). Both terminal COs are redshifted by 9 cm^−1^, whereas the bridging µCO is redshifted by 7 cm^−1^ compared to HydA1-PDT H_red_ (see Table 1). The cyanide ligand vibration frequencies are lowered by 3–4 cm^−1^. The small redshifts in HydA1-PDSe from the oxidized to the reduced state indicate that the reduction takes place at the [4Fe–4S]_H_ subcluster leading to [4Fe–4S]_H_^+^ HydA1-PDSe with a [Fe_p_(I)Fe_d_(II)] configuration. A reduction at the [2Fe]_H_ subsite would lead to larger shifts, e.g., as can be observed in the sensing hydrogenase HydS-ADT from *Thermotoga maritima* upon conversion from the H_ox_ into the H_red*_ state [39].

**Table 1:** FTIR vibrations in cm^−1^ of HydA1-PDT and -PDSe in their oxidized and reduced states

	CN^−^	CO	CO	µCO
PDT H_ox_	2090/2073	1966	1942	1811
PDSe H_ox_	2087/2069	1958	1934	1803
PDT H_red_	2085/2066	1964	1935	1799
PDSe H_red_	2081/2062	1955	1926	1792

These observations indicate that the two enzyme variants have a similar charge distribution with only minor effects caused by the larger mass of selenium as compared to sulfur.

#### EPR spectroscopy

The singly reduced states H_red_ in HydA1-PDT and -PDSe are EPR silent, although both the reduced [4Fe–4S]_H_ and mixed valence [2Fe]_H_ subclusters formally carry unpaired spin density. The anti-ferromagnetic intercluster spin coupling, however, leads to an S_total_ = 0 ground state.

**Fig. 3:**
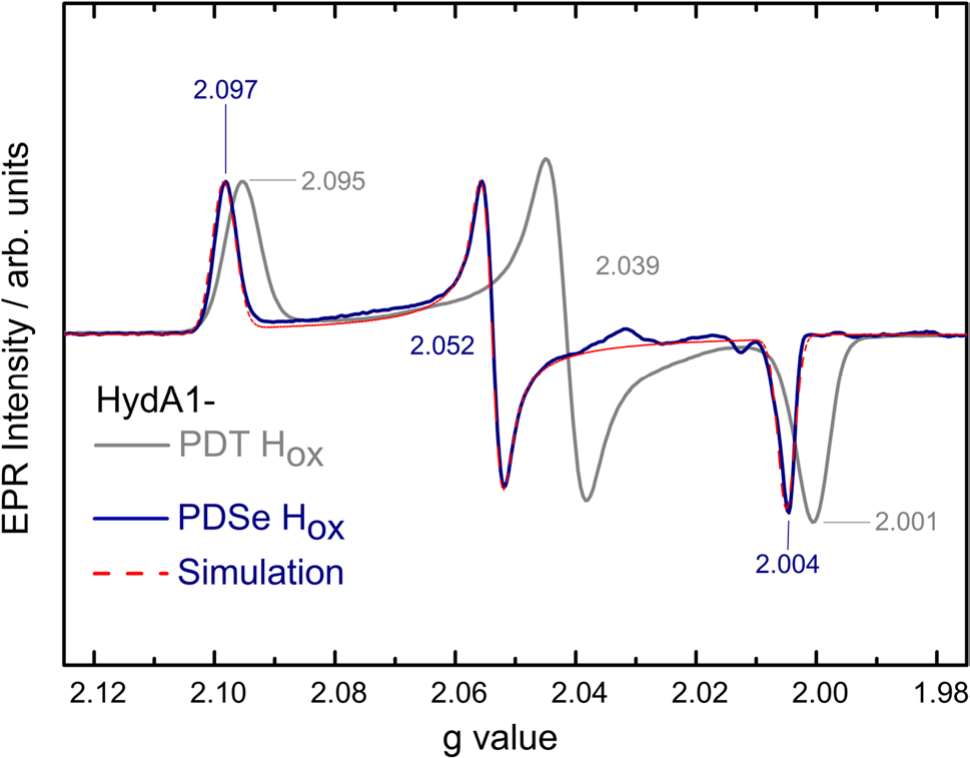
EPR spectra of HydA1-PDT H_ox_ (gray) and HydA1-PDSe H_ox_ (blue). Simulation for HydA1-PDSe H_ox_ is shown in red. The small additional signals between *g* = 2.01 and *g* = 2.03 in HydA1-PDSe H_ox_ originate from a contribution of H_ox_–CO (see table S2). Spectra are taken at 20 K

The oxidized HydA1-PDSe was also analyzed with EPR spectroscopy. The H_ox_ state is expected to have an electronic configuration [4Fe–4S]_H_^2+^-[Fe_p_(I)Fe_d_(II)] which has an S = 1/2 ground state. It indeed shows a rhombic EPR spectrum very similar to that of HydA1-PDT H_ox_ (see Fig. 3). The *g* values of HydA1-PDSe are, however, significantly shifted toward higher values (in particular g_y_) which can be explained by the larger spin–orbit contribution of selenium as compared to sulfur.

#### ^1^H NMR spectroscopy

**Fig. 4:**
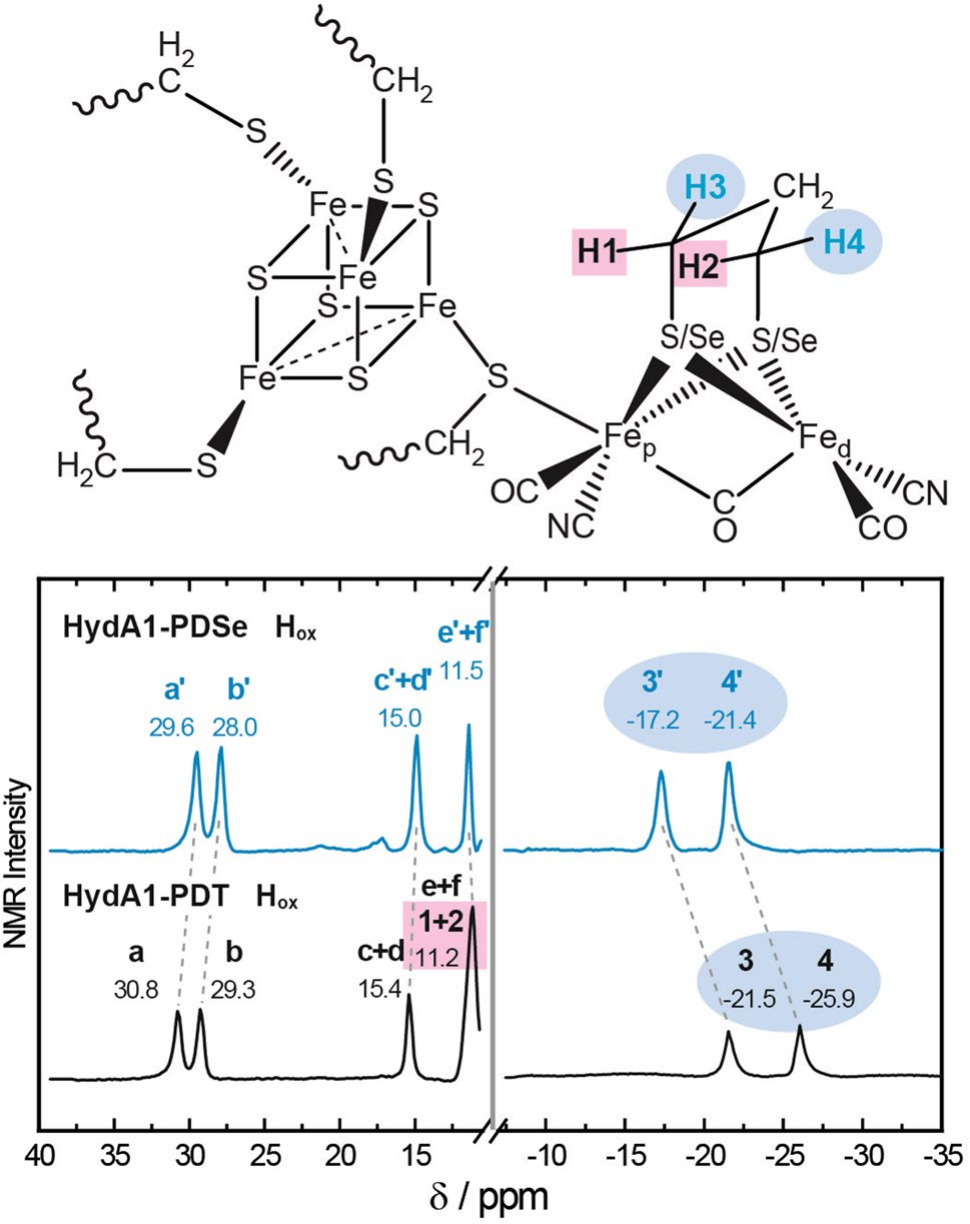
Solution state 600 MHz ^1^H NMR spectra for HydA1-PDT (gray)/-PDSe (blue) in the H_ox_ state at room temperature. Nomenclature used is based on Rumpel et al. [26]. Resonances **a–f/a′–f′** originate from the methylene protons of the cysteines coordinating the [4Fe–4S]_H_ subcluster, while resonances **1–4/1′–4′** are associated with the methylene protons of the PDT/PDSe bridging ligand as indicated in the scheme (top)

^1^H NMR spectroscopy can provide information about the electron spin density delocalization of the H-cluster in two otherwise structurally similar enzymatic states. For the measurements, usually performed at room temperature, a stable redox state of the sample is required as was shown for H_ox_ and H_ox_–CO state of the native HydA1 [26]. In contrast to HydA1-ADSe, HydA1-PDSe is very stable in the H_ox_ state and can be used for ^1^H NMR solution studies at room temperature to investigate the effect of selenium substitution. Figure 4 shows the ^1^H NMR spectra of the oxidized states of HydA1-PDT and HydA1-PDSe in comparison. The characteristic contact-shifted signals originate from the methylene protons of the PDT/PDSe bridge and the β-CH_2_ protons of the cysteines ligating the [4Fe–4S]_H_ cluster (see fig. S3) as recently described by Rumpel et al. [26].

Both spectra show a very similar pattern with four downfield and two upfield shifted resonances (see Fig. 4). The two upfield (negative shift) resonances **3 + 4**, and also **3**′** + 4**′, originate, based on the earlier assignment achieved for HydA1-ADT H_ox_ [26], from the methylene protons of the PDT/PDSe bridge pointing away from the [4Fe–4S]_H_ subcluster (H_equatorial_, see fig. S3). The downfield shifted resonances (**a**–**f**) and (**a**′–**f**′) can be assigned to the methylene protons of the β-CH_2_ protons of the cysteines coordinating the [4Fe–4S]_H_ subcluster [26].

In comparison to HydA1-PDT H_ox_, the equatorial methylene protons **3**′** + 4**′ of HydA1-PDSe H_ox_ are shifted by approximately + 4.5 ppm. The axial protons **1 + 2** pointing toward the [4Fe–4S]_H_ cluster resonate around + 11.2 ppm in HydA1-PDT [26], overlapping with the peak of the β-CH_2_ pair **e + f** resulting in an increased signal width of 400 Hz (see also table S1). The smaller linewidth of the **e**′** + f**′ feature (200 Hz) suggests that the **1**′** + 2**′ feature (originating from the axial PDSe methylene protons) is no longer overlapping and probably located within the diamagnetic envelope, i.e., the region between − 1 and + 10 ppm (not shown) where the protons of the diamagnetic part of the protein resonate. The smaller shifts in HydA1-PDSe show that the protons experience a reduced spin density at the [2Fe]_H_ subsite as well as in the [4Fe–4S]_H_ cluster. The downfield resonances **a**–**f** from HydA1-PDT H_ox_ are assigned to the β-methylene protons of the cysteine side chains coordinating the [4Fe–4S]_H_ cluster. In HydA1-PDSe H_ox_, the β–CH_2_ protons (**a**′–**f**′) are only slightly shifted by approximately − 0.5 to − 1 ppm compared to HydA1-PDT H_ox_.

The electron spin state and the distance of the observed nucleus to the iron in combination with the spin density at the nucleus is correlated with the broadening of its magnetic resonance signal [40]. The similarity in signal width between HydA1-PDT H_ox_ and HydA1-PDSe H_ox_ (see table S1) indicates that there is no difference in spin state and spin density distribution.

For the reduced proteins, the obtained ^1^H NMR spectra in solution show more proton signals over an extended chemical shift range (80 to − 25 ppm) with broader line widths due to a reduced cubane cluster with S = 1/2 (see fig. S4). Although the [4Fe–4S]_H_ cluster is coupled with the [2Fe]_H_ subsite to an EPR silent H_red_ state at low temperatures, at room temperature higher spin states are populated that induce the large chemical shifts. The data are collected and compared in table S1.

#### FTIR spectroelectrochemistry

As inferred from the magnetic resonance and FTIR experiments, the substitution of sulfur to selenium in the bridging position slightly changes the electronic structure of the altered active site bringing more charge density to the [2Fe]_H_ subsite. The small redshift of the FTIR pattern indicates that the redox reaction takes place at the [4Fe–4S]_H_ cluster.

**Fig. 5:**
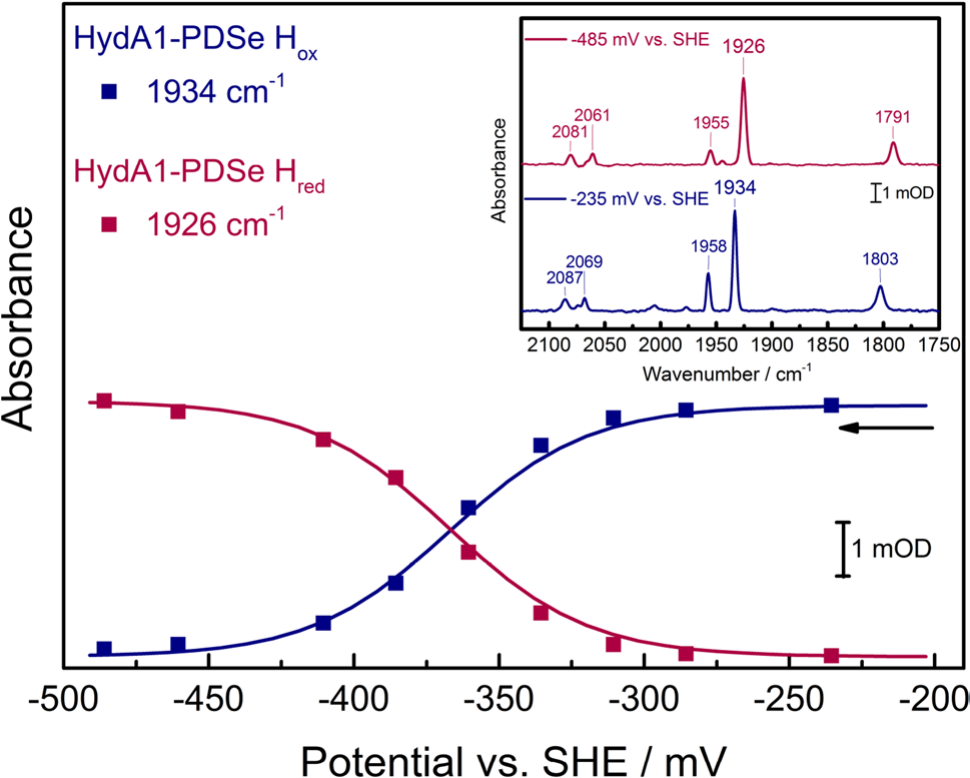
Reductive titration of HydA1-PDSe monitored by FTIR spectroelectrochemistry with selected FTIR spectra. Blue squares represent the intensities of the marker band for the H_ox_ state and red squares for the H_red_ state (see inset). Data are collected at 278 K with 2 cm^−1^ resolution. Solid lines correspond to Nernstian fits with *n* = 1 and give a midpoint potential of *E*_ox/red_ = − 367 ± 20 mV vs SHE; the arrow indicates the titration direction

To analyze the redox properties of the [4Fe–4S]_H_ subcluster in HydA1-PDSe in detail, FTIR spectroelectrochemistry was performed to determine the midpoint potential of the ox/red transition (see Fig. 5). The redox titration of HydA1-PDSe shows the main transition from H_ox_ to H_red_. The plotted absorbance of the two chosen marker bands 1934 and 1926 cm^−1^ against the applied potential are fitted with one electron Nernstian curves. They give a midpoint potential of − 367 ± 20 mV vs SHE, which is 22 mV lower than that of HydA1-PDT [23]. Although this difference is small, a slightly more negative redox potential is consistent with the increased electron density at the [2Fe]_H_ core [19] which is coupled to the cubane cluster.

### The H_ox_–CO state

In contrast to HydA1-PDT, a CO inhibited state is formed under CO gas exposure in HydA1-PDSe. While in HydA1-ADT, the CO inhibited state occurs both in oxidized (H_ox_–CO) and reduced form (H_red_–CO), the CO bound state in HydA1-PDSe can only be stabilized in the oxidized state (see fig. S2A) and will therefore be compared to HydA1-ADT H_ox_–CO.

#### FTIR spectroscopy and FTIR spectroelectrochemistry

**Fig. 6:**
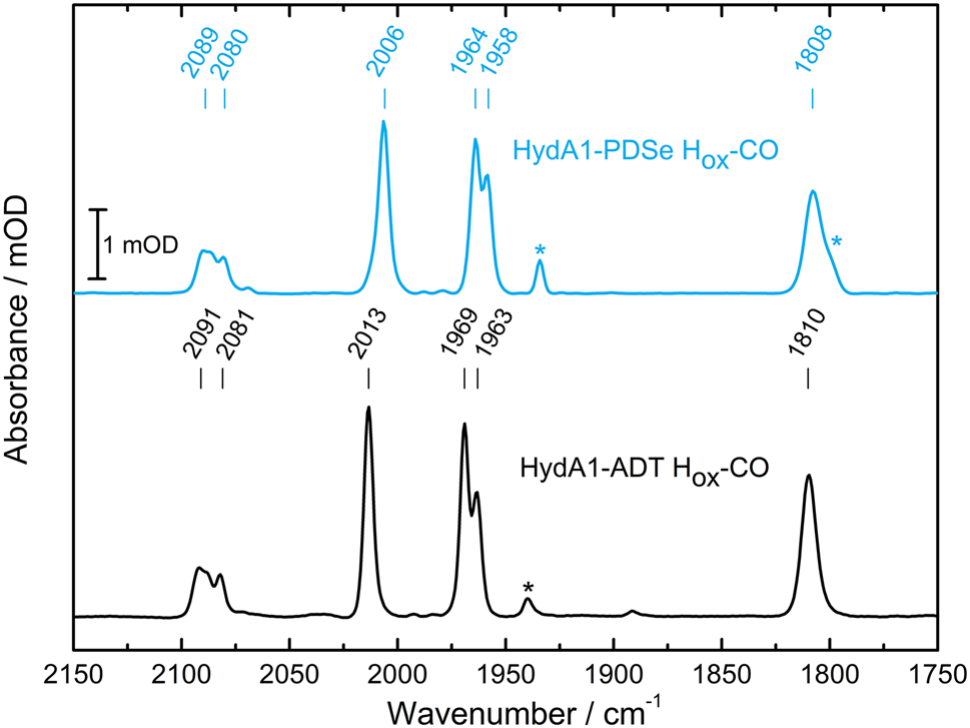
Comparison of the FTIR spectra for the H_ox_–CO states of HydA1-ADT and HydA1-PDSe. The asterisks indicate minor contributions from the H_ox_ state in the samples. Spectra are taken at 288 K with 2 cm^−1^ resolution

The FTIR spectra of the two H_ox_–CO states show the same peak pattern (see Fig. 6). In comparison to HydA1-ADT H_ox_–CO (2012, 1972, 1964 cm^−1^), the FTIR vibrations of the CO ligands in HydA1-PDSe H_ox_–CO state (2006, 1964, 1958 cm^−1^) are slightly red shifted as for the H_ox_ state.

Reductive treatment (2 mM NaDT) of HydA1-PDSe H_ox_–CO partly converts it into the H_red_ state with simultaneous FTIR band broadening from ≈ 6 to ≈ 15 cm^−1^ (FWHM) indicating a partial detachment from the protein scaffold (see fig. S1 A1–A4). Due to the lack of a reduced product from the H_ox_–CO state, the corresponding FTIR spectroelectrochemistry (see fig. S6) shows only vibrational peaks decreasing in intensity during reduction. This behavior was also observed for the native [FeFe] hydrogenase from *D.* *desulfuricans* [41].

In native HydA1 from *C.* *reinhardtii*, however, the H_ox_–CO state is readily reduced to a pure H_red_–CO state at − 470 mV (pH 8.0) [23]. Since the apparent midpoint potential for HydA1-PDSe H_ox_–CO reduction (approximately, − 337 mV, see fig. S6) is even more positive than *E*_ox/red_ (corresponding to the H_ox_/H_red_ transition) for HydA1-PDSe − 367 mV, see Fig. 5, we must conclude that the CO inhibited state of HydA1-PDSe is much less stable than that in the native enzyme.

#### EPR spectroscopy

**Fig. 7:**
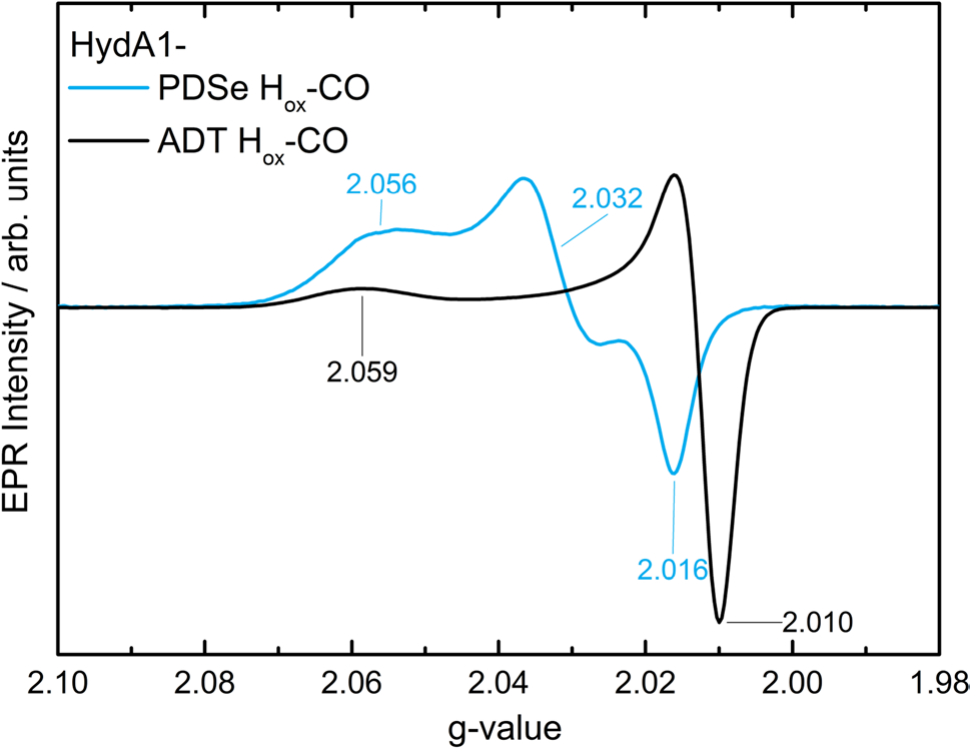
Superimposed CW X-band EPR spectra of HydA1-ADT H_ox_–CO and HydA1-PDSe H_ox_–CO. Spectra are recorded from samples with 1 mM protein concentration at 20 K

Since HydA1-PDT is not inhibited by CO, the native enzyme is again used for comparison. HydA1-PDSe H_ox_–CO gives a rhombic EPR spectrum with rather broad lines, whereas the known HydA1-ADT H_ox_–CO state is characterized by an axial EPR spectrum with narrower lines [42] (see Fig. 7). Two of the g-values are significantly shifted toward higher values which, as for the HydA1-PDSe H_ox_ state, can be explained by the larger spin–orbit contribution of selenium compared to sulfur. Thus, the EPR spectrum suggests that the electronic structure of the iron core in HydA1-PDSe H_ox_–CO deviates significantly from that in the native H_ox_–CO state.

### Reconstitution of apo-HydA1 with [4Fe–4Se]_H_

Owing to the possibility of heterologous expression, the cubane cluster of apo-HydA1 could also be reconstituted with selenium (see “Materials and methods”) leading to a stable [4Fe–4Se]_H_ hydrogenase which was analyzed with EPR and NMR spectroscopy.

#### ^1^H NMR spectroscopy

**Fig. 8:**
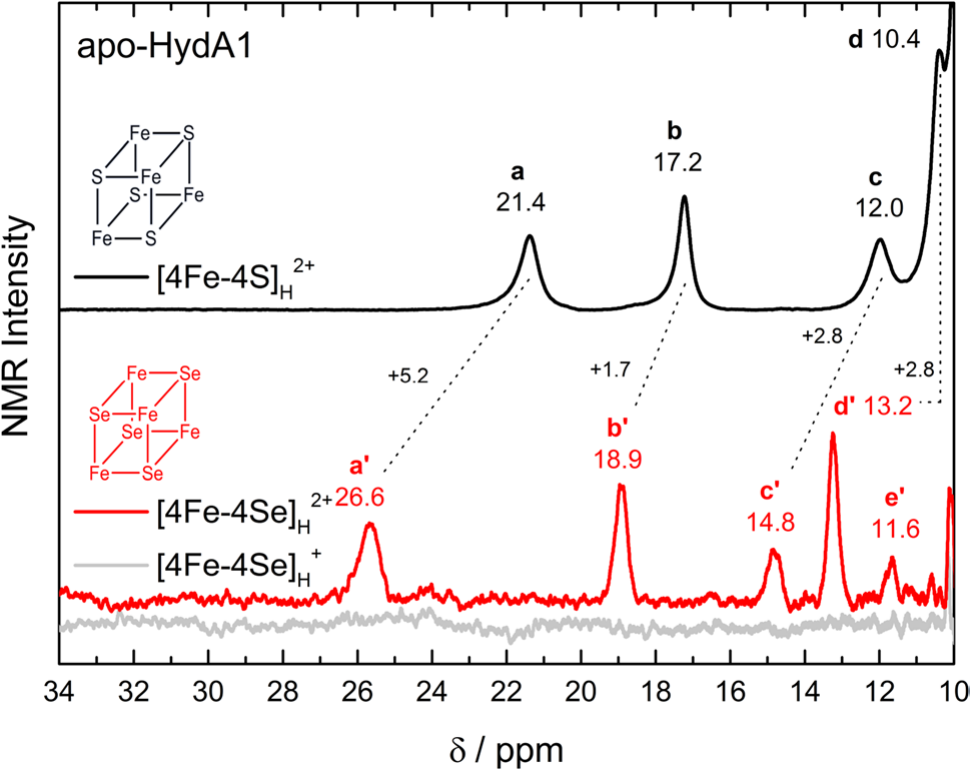
Scaled 600 MHz ^1^H NMR spectra of apo-HydA1 comparing the native [4Fe–4S]_H_ with the reconstituted [4Fe–4Se]_H_ cluster. Protein concentrations were 1.7 mM and 0.8 mM, respectively. For the apo-HydA1 with the [4Fe–4S]_H_ cluster, only the oxidized state (black) and for the selenium substituted enzyme the oxidized (red) and reduced state (gray) are shown in the 34–10 ppm range. Spectra are recorded at 298 K

The ^1^H NMR spectra of apo-HydA1 containing either the native [4Fe–4S]_H_ or the [4Fe–4Se]_H_ subcluster in their oxidized (2 +) state show a similar pattern of the contact-shifted β-CH_2_ proton resonances (**a**–**d** and **a**′–**e**′) in the downfield region. They originate from the Fe coordinating cysteines confirming a successful reconstitution with no significant difference between the two structures (see Fig. 8). In the oxidized state of apo-HydA1 with the [4Fe–4S]_H_ cluster, the two S = 9/2 Fe(II)Fe(III) pairs in the cubane cluster are antiferromagnetically coupled to form a diamagnetic S = 0 ground state. At room temperature, paramagnetism arises from population of the excited states [43], explaining the anti-Curie temperature dependence of the ^1^H chemical shifts [26]. Sulfur-to-selenium exchange in the cubane cluster increases the chemical shift of the hyperfine shifted proton resonances in the range from 2.8 to 5.2 ppm, being indicative of a stronger magnetic interaction. The overall larger chemical shifts in the [4Fe–4Se]_H_ substituted enzyme uncover an additional proton signal at 11.6 ppm, labeled **e**′.

For the dithionite reduced cubane cluster, the effect of Se substitution is dramatic. Whereas the $\left[ { 4 {\text{Fe}} - 4 {\text{S}}} \right]_{\text{H}}^{ + }$ cluster shows a classical S = 1/2 ground state with broad resonances occurring over a more extended field range (down to 55 ppm) [26], the $\left[ { 4 {\text{Fe}} - 4 {\text{Se}}} \right]_{\text{H}}^{ + }$ apo-HydA1 seems to be in a high spin state with ^1^H NMR features that are broadened beyond detection (see gray trace Fig. 8). For small bacterial ferredoxins (≈ 6 kDa), selenium substitution also results in high electron spin states. But here, paramagnetically shifted resonances in the range from − 45 to + 160 ppm could be observed without extreme broadening. This can be explained by taking into account the eight times smaller size of the ferredoxins as compared to apo-HydA1 [27].

#### EPR spectroscopy on reduced [4Fe–4Se]_H_ apo-HydA1

EPR spectroscopy can contribute to understanding the lack of ^1^H NMR signals introduced by high electron spin states in combination with the large size (≈ 50 kDa) of [4Fe–4Se]_H_ apo-HydA1.

**Fig. 9:**
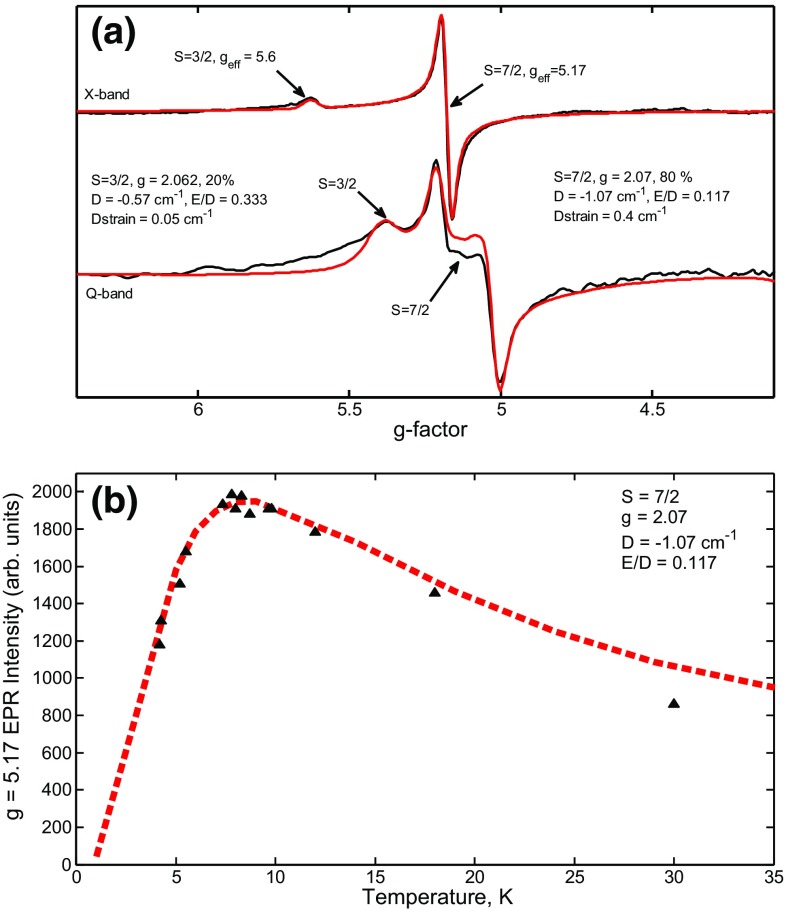
CW X-band and Q-band EPR of reduced [4Fe–4Se]_H_ apo-HydA1. **a** CW X- and Q-band EPR spectra recorded at 10 K (black) with corresponding simulations (red) showing S = 3/2 and S = 7/2 species. **b** Temperature dependence of the signal at *g* = 5.17; amplitudes were recorded at X-band frequency with corresponding fit to determine the zero field splitting parameter *D*

The EPR spectra of the reduced [4Fe–4Se]_H_ apo-HydA1shown in Fig. 9a with EPR features at *g* = 5.17 and *g* = 5.6 are indicative of a mixed high spin state. These high spin states have been described earlier for Se-substituted ferredoxins [30, 32, 33]. The derivative feature at *g* = 5.17 is assigned to the excited S = 3/2 Kramers doublet of the S = 7/2 ground state assuming a rhombicity of *E*/*D* = 0.117 [44]. The temperature dependence of the amplitude of this feature is consistent with a *D* = − 1.07 cm^−1^ (see Fig. 9b). The absorptive feature at *g* = 5.63 is assigned to anisotropic components of both S = 1/2 and S = 3/2 Kramers doublets of an S = 3/2 ground state [33]. It is assumed that the occurrence of high spin components of the Se-substituted cubane cluster is related to its coordination environment which is modulating the intra-cluster exchange coupling(s).

Subtle changes of the protein coordination to the [4Fe–4Se] cluster as affected by protein–protein binding interactions in photosystem I (PSI) have led to a spin crossover [33]. We observed a similar effect when the [4Fe–4Se]_H_ apo-HydA1 enzyme was maturated with [2Fe]–ADT/–PDT/–PDSe. The familiar S = 1/2 species of the H_ox_ state can be identified (see fig. S6). At minimum, this shows that the oxidized [4Fe–4Se]^2+^ cluster is in a low spin configuration. The ^1^H NMR spectrum of the [4Fe–4Se]_H_ HydA1-PDT H_red_ state in figure S4 shows paramagnetic shifts very similar to the [4Fe–4S]_H_ HydA1-PDT H_red_ state. This indicates that the reduced $\left[ { 4 {\text{Fe}} - 4 {\text{Se}}} \right]_{\text{H}}^{ + }$ cluster in HydA1-PDT H_red_ is also in a low spin state (formally, S = 1/2) and couples antiferromagnetically to the S = 1/2 state of the [2Fe]_H_ binuclear subcluster to generate a diamagnetic ground state. Further changes are not observed for the [4Fe–4Se]_H_ HydA1 maturated samples as expected from the previous results of Noth et al. [7]. (see fig. S6 and S7).

## Summary and conclusions

In this work, we have shown that S-to-Se substitution in the bridging ligand of the binuclear subsite in the H-cluster of the [FeFe] hydrogenase from *C. reinhardtii* induces distinct changes in the electronic structure of the enzyme’s active site. According to the FTIR signatures of the H_ox_ and H_red_ states, the charge density on the [2Fe]_H_ site in HydA1-PDSe is increased. Part of the charge density is transported onto the [4Fe–4S]_H_ subcluster explaining the slightly lower midpoint potential of the H_ox_/H_red_ transition. The increased charge density lowers the reduction potential and may also contribute to the increased oxygen sensitivity of the active *Cp*HydA1-ADSe observed in an earlier study [22].

The observed reduced paramagnetic shift of the methylene protons in the bridging ligand in ^1^H NMR spectroscopy shows that less spin density remains on the PDSe ligand than on the PDT ligand. The increased size of selenium with respect to sulfur causes the Fe–Se bond to be somewhat longer than the corresponding Fe–S bond. This reduces the steric bulk of the CH_2_ bridgehead at the open coordination site and may be a contributing factor allowing an external CO to bind at Fe_d_ in HydA1-PDSe in contrast to the situation in HydA1-PDT. The extraneous CO ligand significantly affects the electronic structure of the [2Fe]_H_ subcluster as is apparent from the rhombic EPR spectrum of HydA1-PDSe H_ox_–CO that strongly deviates from that of HydA1-ADT H_ox_–CO. Although the reduced steric bulk of the –CH_2_ bridgehead allows formation of the H_ox_–CO state, it still destabilizes this state sufficiently such that the HydA1-PDSe H_ox_–CO state cannot be reduced without detaching the extraneous CO or the [2Fe]_H_ subsite as a whole from the H-cluster [41].

The effect of substituting selenium for the inorganic sulfides in the [4Fe–4S]_H_ cluster is different from the effect of PDT to PDSe substitution in the [2Fe]_H_ subsite. In the oxidized [4Fe–4Se]^2+^ protein, more spin density is transported to the Fe ions leading to large chemical shifts in the NMR spectrum. In the reduced [4Fe–4Se]^+^ protein, the spin coupling between the iron ions becomes critically dependent on the first coordination sphere and leads to a mixed spin state of S = 3/2 and S = 7/2. Maturation of the [4Fe–4Se]^2+^ apoprotein, however, restores the coordination environment to that of the native enzyme resulting in a low spin configuration with S = 1/2. The FTIR patterns of the [4Fe–4Se]_H_ HydA1-PDT/-PDSe samples do not differ from the [4Fe–4S]_H_ cluster-containing samples.

The discussed effects of S → Se substitution on spectroscopy and activity of the [FeFe] hydrogenase HydA1 illustrate the intricate balance of spin interactions and charge density distributions within the H-cluster governing the electronic structure and catalytic behavior of the enzyme’s active site.

## Electronic supplementary material

Below is the link to the electronic supplementary material.

Supplementary material 1 (PDF 1215 kb)

## Abbreviations

MV: Methyl viologen
NMR: Nuclear magnetic resonance
EPR: Electron paramagnetic resonance
FTIR: Fourier transform infrared spectroscopy

## Bridging ligands

ADT: Aza-propane-dithiolate (µ(SCH_2_)_2_NH)
ADSe: Aza-propane-diselenate (µ(SeCH_2_)_2_NH)
PDT: Propane-dithiolate (µ(SCH_2_)_2_CH_2_)
PDSe: Propane-diselenate (µ(SeCH_2_)_2_CH_2_)

## Synthetic precursors

[2Fe]–ADT/–ADSe/–PDT/–PDSe: [2Fe] = Fe_2_(CO)_4_(CN^−^)_2_

## Enzymes

apo-HydA1: *Cr*HydA1 containing only the [4Fe–4S/Se]_H_ subsite
HydA1-ADT/-PDT: *Cr*HydA1 maturated with [2Fe]-ADT or [2Fe]-PDT
*Cr*: *Chlamydomonas reinhardtii*

## Subsites

[2Fe]_H_: Fe_2_(CO)_3_(CN^−^)_2_ADT/ADSe/PDT/PDSe, subsite of [FeFe] hydrogenase
