# Supplementary material for: Spectroscopic investigations of a semi-synthetic [FeFe] hydrogenase with propane di-selenol as bridging ligand in the binuclear subsite: comparison to the wild type and propane di-thiol variants
Source: J Biol Inorg Chem. 2018 Apr 7;23(3):481–91. doi: 10.1007/s00775-018-1558-4 (PMC5940705; doi:10.1007/s00775-018-1558-4)
Supplement: Supplementary file 1 — Supplementary material 1 (PDF 1215 kb) [file 775_2018_1558_MOESM1_ESM.pdf]

Supporting Information

Spectroscopic investigations of a semi-synthetic [FeFe] hydrogenase with propane di-selenol as bridging ligand in the binuclear subsite. Comparison to the wild type and propane di-thiol variants.

C. Sommer[1], S. Rumpel[1], S. Roy[2], C. Farès[3], V. Artero[2], M. Fontecave[4], E. Reijerse[1], W. Lubitz[1]

1 Max-Planck-Institut für Chemische Energiekonversion, Stiftstrasse 34-36, 45470 Mülheim an der Ruhr, Germany.

2 Laboratoire de Chimie et Biologie des Métaux, Université Grenoble Alpes, CEA/BIG, CNRS, 17 rue des martyrs, 38000 Grenoble, France.

3 Max-Planck-Institut für Kohlenforschung, Kaiser-Wilhelm Platz 1, 45470 Mülheim an der Ruhr, Germany

4 Laboratoire de Chimie des Processus Biologiques, Collège de France, Université Pierre et Marie Curie, CNRS UMR 8229, PSL Research University, 11 place Marcelin Berthelot, 75005 Paris, France.

✉ Wolfgang.Lubitz@cec.mpg.de

1. Catalytic activity of HydA1-PDSe as compared to that of HydA1-PDT

Although HydA1 incorporates the [2Fe]-PDSe complex no activity could be measured, neither in hydrogen production nor in hydrogen oxidation (see fig.S1). For HydA1-PDT a residual activity in both tests can be measured that are 0.49 H_2/s for H_2 production and 0.06 H_2/s for H_2 consumption, which is less than 1% of WT activity. HydA1-PDSe shows no activity.

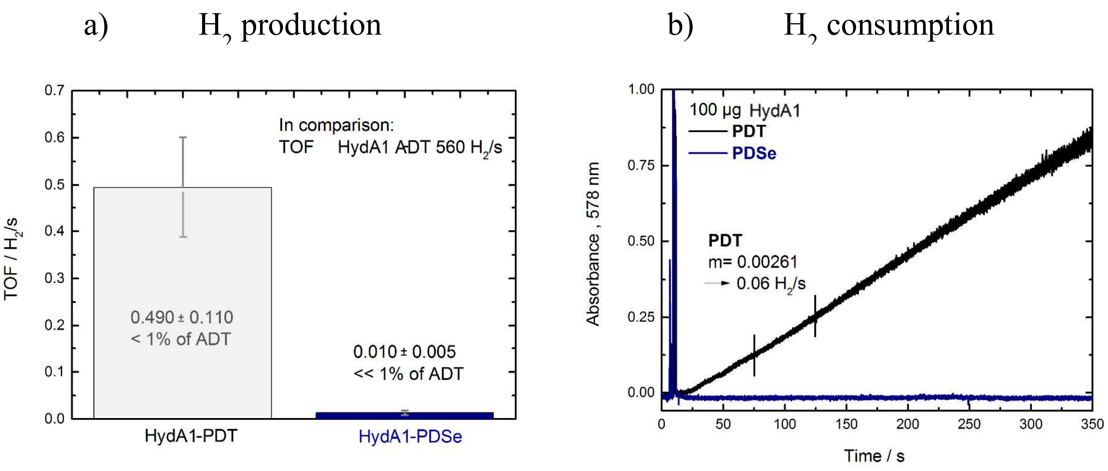

Fig. S1 Activities of H_2 production and H_2 consumption for HydA1-PDT/-PDSe. a) H_2 production was measured with GC analysis after incubation of 10 µg protein for 20 min at 310 K with 10 mM MV/100 mM NaDT. b) H_2 consumption was measured with 100 µg protein, 10 mM MV and hydrogen saturated buffer at room temperature

2. Maturation of apo-HydA1 with $[Fe_2(PDSe)(CO)_4(CN)_2)]^{2-}$

FTIR spectroscopy was used to demonstrate that the [2Fe]-PDSe cofactor was incorporated into the protein scaffold. The spectrum of the free complex in aqueous solution is shown in figure S2 part B and is characterized by its three broad CO bands (1972/1943/1906 cm^{-1}) and an even broader contribution in the region of the CN^- bands (2050 cm^{-1}). This fingerprint pattern is very similar to the one observed for free [2Fe]-PDT.[1] With ^{15}N labeled CN^-

ligands a wavenumber shift of -30 cm^{-1} occurs for the CN^- bands while the CO peaks are only slightly shifted indicating weak vibrational coupling to the CN^- ligands. No additional changes occur. Figure S2B shows the FTIR spectrum obtained after maturation of apo-HydA1 with [2Fe]-PDSe and exposure to CO. The clear band narrowing to 6-8 cm^{-1} indicates successful incorporation of the binuclear cofactor into the hydrogenase.

Additionally, figure S2 shows how the active site is disassembled by trying to reduce the H_{ox}-CO state (A2-A4). With minor amounts of dithionite (DT) the reduced state H_{red} (1926/1792 cm^{-1}) and peaks related to an unknown species appear. Already with 0.5 mM DT all H_{ox}-CO peaks disappeared. With 10 mM DT only broad, unidentified contributions are detected that most likely arise from partly detached [2Fe]-PDSe.

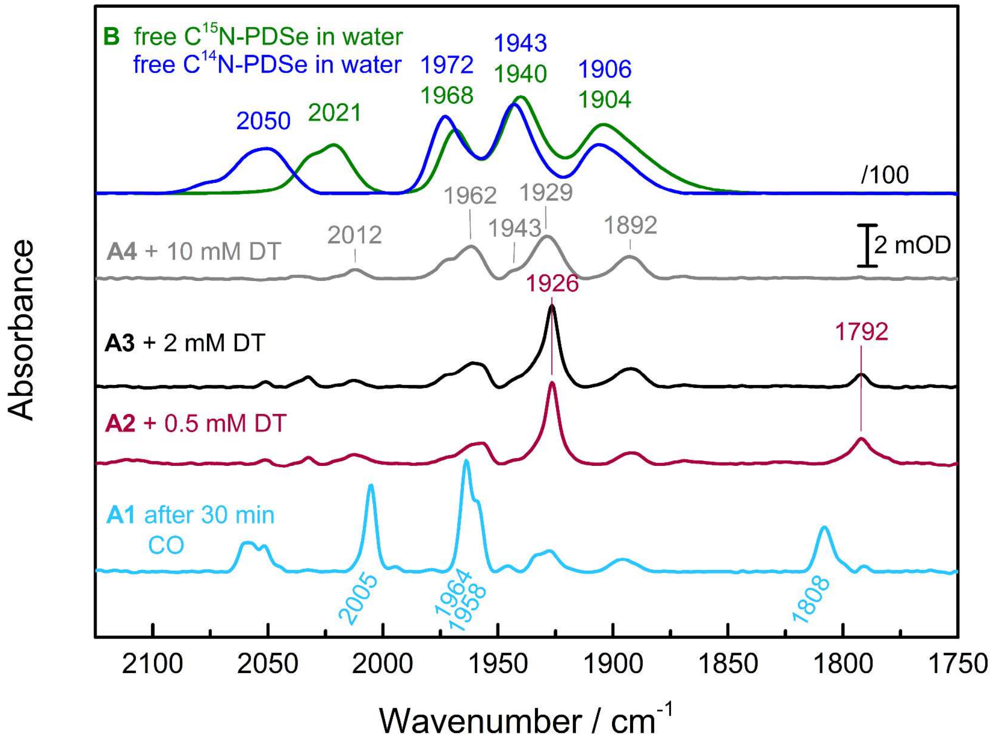

Fig. S2 FTIR spectra of HydA1-C^{15}N-PDSe in the H_{ox}-CO state (light blue) with reductive titration and a comparison with the free complexes dissolved in water. Spectra labeled with A show HydA1-C^{15}N-PDSe after 30 min exposure to 1 bar CO(g) (A1) and addition of different concentrations of dithionite (A2-A4). Superimposed FTIR spectra in B

show the $C^{15}N$ labeled free complex compared to the free complex with $C^{14}N$ in aqueous buffer (0.1 M TRIS, 0.15 M NaCl, pH 8.0). Spectra are taken at 288 K with a resolution of 2 cm^{-1}

3. ^{1}H NMR of HydA1-PDSe H_{ox}/H_{red} in comparison to HydA1-PDT

In the 3D structure of the active site (see fig.S3) the difference of the methylene proton pairs in the bridge become obvious. The axial oriented protons (H_{ax}) are located above Fe_p with a distances of about 3.44 Å. The equatorial protons (H_{eq}) have a distance to Fe_p of ≈4.37 Å.[2] These distances are of interest because in the H_{ox}-CO state the spin density is assumed to be located at Fe_p.[3]

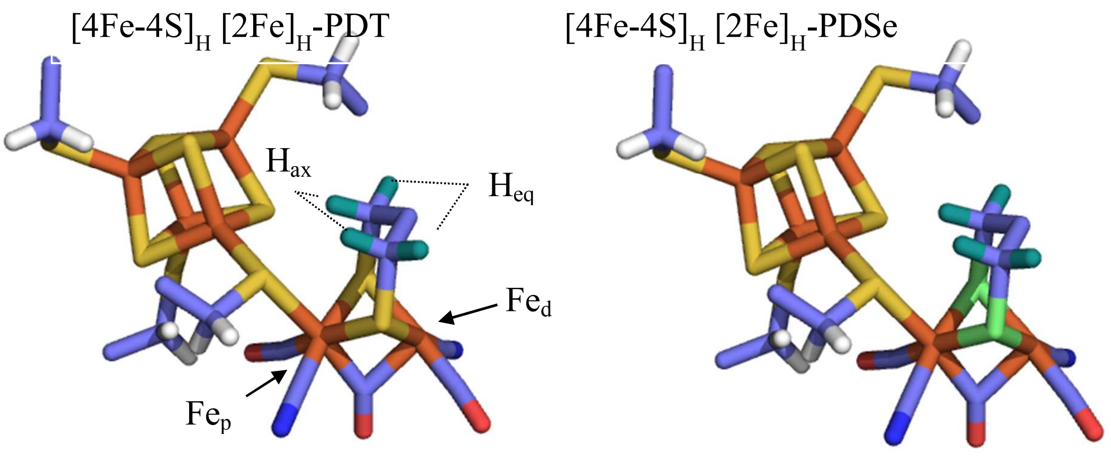

Fig. S3 H-Cluster of PDT (left) and PDSe (right) maturated [FeFe] hydrogenase with β-CH_2 protons (white) of Fe ligating cysteines and methylene protons (aqua blue) of the bridge. The figure was made using PYMOL with PDB 3C8Y[4]. Note that the figure shows the originally –NH bridgehead but colored as carbon to indicate –CH_2. Color code: white β-CH_2 protons, purple: C, yellow: S, orange: Fe, blue: O, red: N, aqua blue: methylene protons in bridge, lime: Se

In the reduced states the resonances of Cys β-CH_2 protons are more downfield shifted and broader than in the oxidized state (62 versus 31 ppm) due to the paramagnetic S=1/2 ground state of the cubane cluster (compare fig.4 and S4).[5] The signal pattern for reduced HydA1-PDT and HydA1-PDSe are very similar and show only a minor effect upon S-to-Se substitution (fig. S4). The upfield shift can be explained by a decreased spin density on the PDSe bridge that affects the methylene protons. The β-CH_2 protons of the ligating cysteines are hardly affected. The $[4Fe\text{-}4Se]_H$ HydA1-PDT H_{red} with its far less shifted Cys β-CH_2 protons follows the described tendency of lower spin density for selenium substituted samples. More interestingly $[4Fe\text{-}4Se]_H$ HydA1-PDT H_{red} shows that even in the reduced state there is no high spin system (for the oxidized state see EPR in fig. S7).

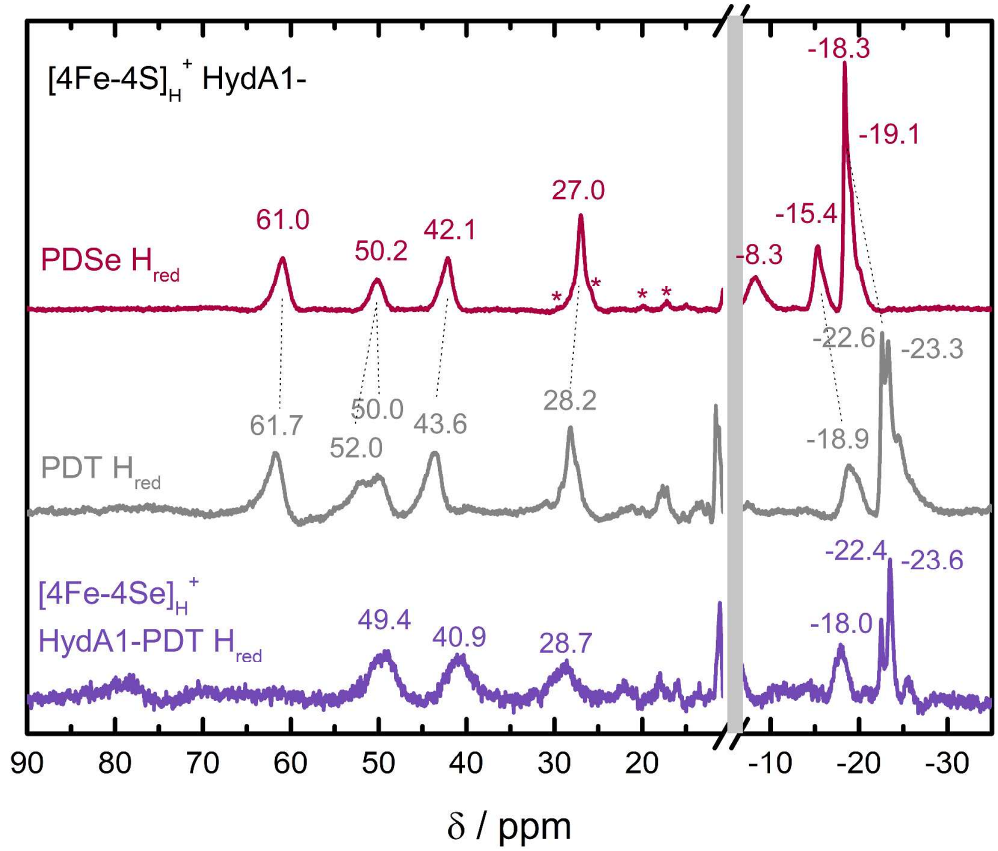

Fig. S4 600 MHz ^{1}H NMR spectra (at 298 K) of HydA1-PDT (grey/purple)/-PDSe (red) in the reduced state. The cubane clusters are reduced (paramagnetic) leading to broader lineshapes. In HydA1-PDSe H_{red} (red) small contributions of the H_{ox} can be seen as

shoulders/little peaks at around 30, 28 and 15 ppm (see asterisks). The downfield region is shown from 90 to 10.8 ppm and the upfield region from -1.5 to -35 ppm

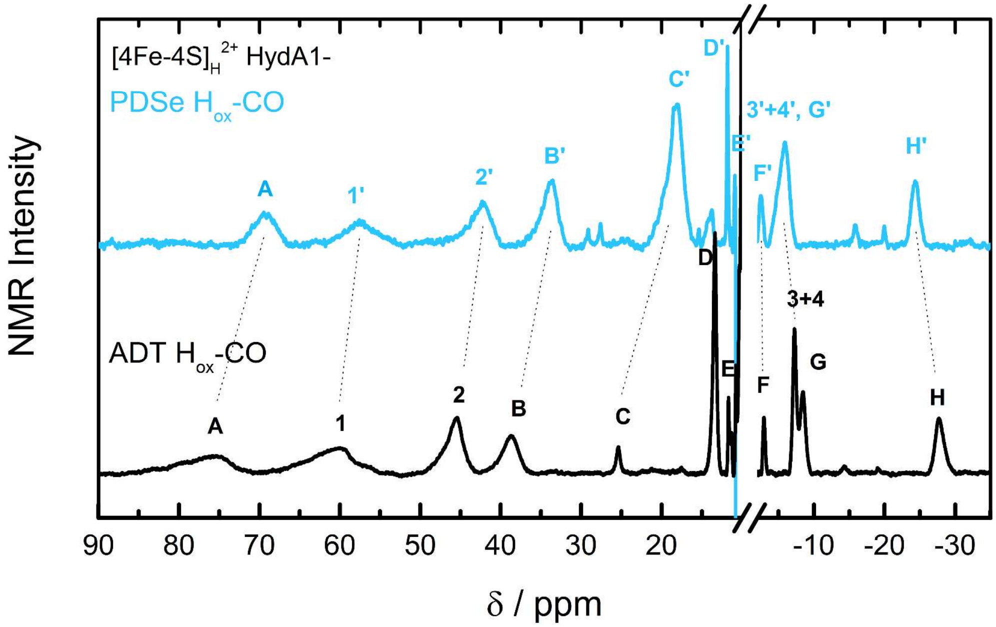

Fig. S5 600 MHz ^{1}H NMR spectra (at 298 K) of HydA1-ADT (black) and HydA1-PDSe (light blue) in the oxidized CO inhibited state. The peak labeling follows the nomenclature in the main text. HydA1-ADT H_{ox}-CO has a concentration about 4 mM, while the HydA1-PDSe H_{ox}-CO sample is about 1.8 mM.

Figure S5 presents a comparison of the ^{1}H NMR spectra of HydA1-PDSe H_{ox}-CO and ADT H_{ox}-CO as a reference system since HydA1-PDT lacks a stable CO-inhibited state. In general, the observed Cys β-CH_2 signals of H_{ox}-CO shows larger downfield and smaller upfield shifts than that of H_{ox} .[2] In the H_{ox} state the spin is distributed more equally over the $[2Fe]_H$ site while in the H_{ox}-CO state it is assumed to be localized at Fe_p[3] which is the reason for the large shift of the axial protons **1**, **2** of the bridge. The labeling of the peaks in HydA1-PDSe H_{ox}-CO is based on homology with the previously studied HydA1-ADT H_{ox}-CO state.[2] For HydA1-PDSe H_{ox}-CO the Cys β-CH_2 protons show smaller downfield shifts

as compared to those observed for HydA1-ADT H_{ox}-CO, suggesting an overall reduction of the spin density at the $[2Fe]_H$ site due to the presence of selenium (see fig. 7).

Table S1: Summary of room temperature 600 MHz ^{1}H NMR data including peak labels, putative assignments, chemical shifts and line widths. Data marked with * are taken from Rumpel *et al*[2], δ = chemical shift. n.d. not determined, n.a not assigned.

Peak label	Assignment	δ / ppm	Width/ Hz	Peak label	Assignment	δ / ppm	Width/ Hz
**[4Fe-4S]$_H$ HydA1-PDT H$_{ox}$**				**[4Fe-4S]$_H$ HydA1-PDSe H$_{ox}$**			
**a**	β-CH_2	30.8	300	**a'**	β-CH_2	29.6	300
**b**	β-CH_2	29.3	300	**b'**	β-CH_2	28.0	300
**c+d**	β-CH_2	15.4	200	**c'+d'**	β-CH_2	15.0	200
**e+f 1+2**	β-CH, H$_{ax}$, H$_{ax}$	11.2	400	**e'+f'**	β-CH_2	11.5	200
**3**	H$_{eq}$	-21.5	360	**3'**	H$_{eq}$	-17.2	310
**4**	H$_{eq}$	-25.9	350	**4'**	H$_{eq}$	-21.4	310
**[4Fe-4S]$_H$ HydA1-ADT Hox-CO***				**[4Fe-4S]$_H$ HydA1-PDSe Hox-CO**			
**A**	β-CH_2	75.3	4000	**A'**	β-CH_2	69.0	1500
**1**	H$_{ax}$	60.0	4000	**1'**	H$_{ax}$	57.3	5400
**2**	H$_{ax}$	45.4	1800	**2'**	H$_{ax}$	42.0	1100
**B**	β-CH_2	38.6	1400	**B'**	β-CH_2	33.7	1400
**C**	β-CH_2	25.4	300	**C'**	β-CH_2	18.0	800
**D**	β-CH_2	13.4	300	**D'**	β-CH_2	14.0	1000
**E**	β-CH_2	11.7	300	**E'**	β-CH_2	11.8	100
**F**	β-CH_2	-2.9	300	**F'**	β-CH_2	-2.5	400
**3+4**	H$_{eq}$	-7.3	300	**3'+4'**	H$_{eq}$	-5.8	700
**G**	β-CH_2	-8.5	400	**G'**	β-CH_2	-5.8	700
**H**	β-CH_2	-27.8	800	**H'**	β-CH_2	-24.4	1000
**[4Fe-4S]$_H$ apo HydA1 oxidized***				**[4Fe-4Se]$_H$ apo HydA1 oxidized**			
**a**	β-CH_2	21.2	300	**a'**	β-CH_2	25.5	400
**b**	β-CH_2	17.2	200	**b'**	β-CH_2	18.9	300
**c**	β-CH_2	12.0	300	**c'**	β-CH_2	14.8	300
**d**	β-CH_2	10.4	200	d'	β-CH_2	13.2	200
				e'	β-CH_2	11.6	200
**[4Fe-4Se]$_H$ HydA1-PDT H$_{ox}$**				**[4Fe-4Se]$_H$ HydA1-PDSe H$_{ox}$**			
	n.a.	32.9	270		n.a.	30.8	270
	n.a.	31.5	290		n.a.	29.3	240
	n.a.	17.0	190		n.a.	15.4	180
	n.a.	15.0	160		n.a.	11.2	400
	n.a.	11.4	330		n.a.	10.3	280
	n.a.	-21.7	330		n.a.	9.6	n.d.
	n.a.	-26.2	300		n.a.	-21.5	350

Peak label	Assignment	δ / ppm	Width / Hz	Peak label	Assignment	δ / ppm	Width/ Hz
**$[4Fe\text{-}4Se]_H$ HydA1-PDT H_{red}**							
	n.a.	49.4	1800				
	n.a.	40.9	1600				
	n.a.	28.7	2000				
	n.a.	-6.00	1100				
	n.a.	-18.0	1000				
	n.a.	-22.4	300				
	n.a.	-23.6	300				
**$[4Fe\text{-}4S]_H$ HydA1-PDT H_{red}**				**$[4Fe\text{-}4S]_H$ HydA1-PDSe H_{red}**			
	n.a.	61.7	1100		n.a.	61.0	1100
	n.a.	52.0	1000		n.a.	50.2	1000
	n.a.	50.0	1000		n.a.	42.1	1000
	n.a.	43.6	1100		n.a.	27.0	900
	n.a.	28.2	900				
	n.a.	17.7	700				
	n.a.	11.7	200				
	n.a.	-1.9	n.d.		n.a.	-1.8	200
	n.a.	-18.9	1100		n.a.	-8.3	800
	n.a.	-22.6	250		n.a.	-15.4	900
	n.a.	-23.3	400		n.a.	-18.3	200
					n.a.	-19.1	200

4. FTIR spectroelectrochemistry of HydA1-PDSe H_{ox}-CO

The reductive potential titration of the H_{ox}-CO state of HydA1-PDSe shows the disappearing of the typical H_{ox}-CO signals but no conversion into a CO inhibited reduced state. Instead, the H_{red} state as well as an unidentified species is formed (see fig. S2 A1-A4).

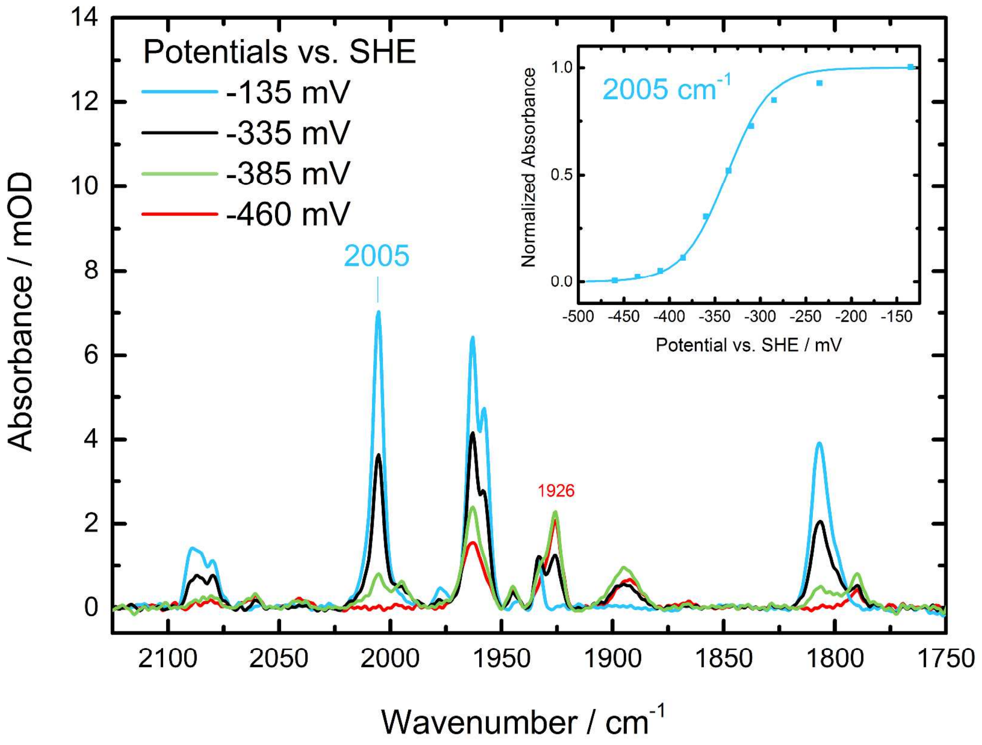

Fig. S6 Selected FTIR spectra of HydA1-PDSe H_{ox}-CO recorded at different applied potentials as indicated by the color code. At the lowest potential (-460 mV) the signature of the H_{ox}-CO state (e.g. the band at 2005 cm^{-1}) has disappeared. The band appearing at 1926 cm^{-1} suggests partial conversion into the H_{red} state. The inset shows the reductive titration using FTIR spectroelectrochemistry of HydA1-PDSe H_{ox}-CO following the normalized marker band for the H_{ox}-CO state at 2005 cm^{-1}. Blue squares represent the (relative) intensities of this marker band. The solid line corresponds to a Nernstian fit yielding a midpoint potential of -337 mV vs. SHE with n=1. Data are collected at 278 K with 2 cm^{-1} resolution

Applying a reductive potential to HydA1-PDSe H_{ox}-CO does not result in a reduced CO-inhibited species but in an overall intensity decrease. Taking into account only the disappearance of the H_{ox}-CO state an apparent midpoint potential of -337 mV vs. SHE can be calculated that is by 130 mV higher than the corresponding midpoint potential for HydA1-ADT.[6] Due to the different composition of the bridging ligand, the properties of the CO bound state of HydA1-PDSe cannot be directly compared to the native enzyme. From our observations one must conclude that the CO bound state in HydA1-PDSe is much less stable than that in the native HydA1-ADT.

5. EPR of $[4Fe\text{-}4Se]_H$ HydA1-ADT, -PDT and -PDSe

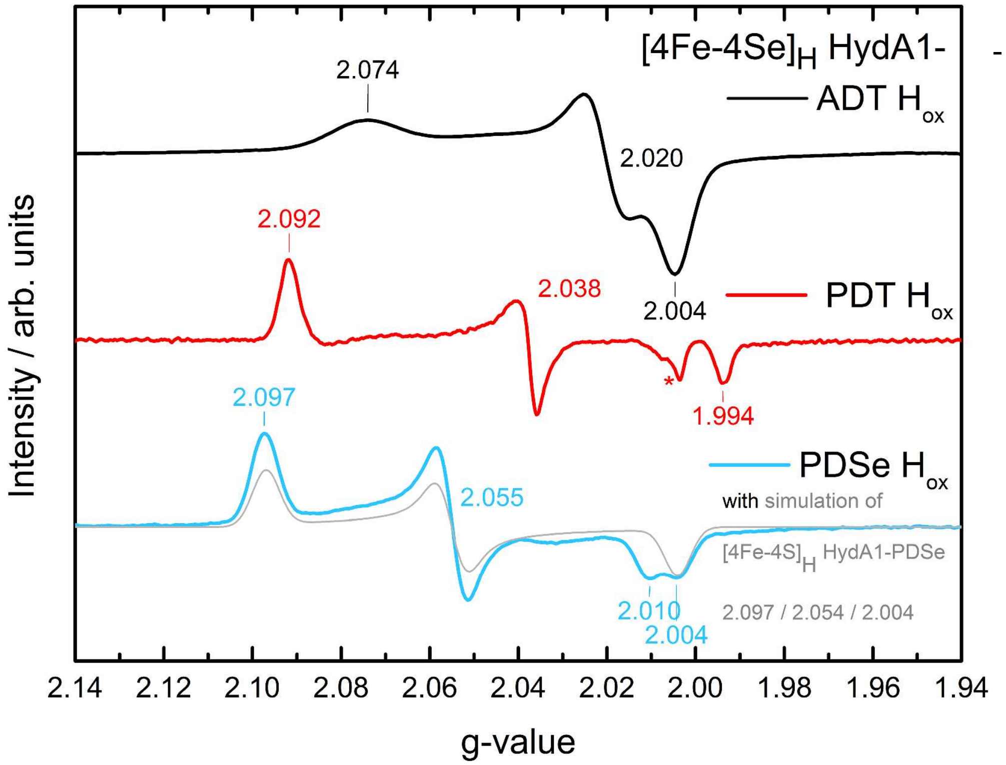

Fig. S7 CW X-band EPR spectra of $[4Fe\text{-}4Se]_H$ HydA1-ADT (black)/-PDT (red)/-PDSe (light blue) at pH 8. The $[4Fe\text{-}4Se]_H$ HydA1-PDSe sample shows a contamination by the sulfur containing cubane $[4Fe\text{-}4S]_H$ HydA1-PDSe as confirmed by the simulation with g-values from fig.3. The additional signals marked with an asterisk in HydA1-PDT H_{ox} are background signals from the resonator

Figure S7 shows the EPR spectra of the $[4Fe\text{-}4Se]_H$ reconstituted enzyme with the native [2Fe]-ADT cofactor as well as with the -PDT and -PDSe variants. All mimics lead to a S=1/2 spin state when the $[4Fe\text{-}4Se]_H$ apo-enzyme was successfully maturated. The differences in g-values between the three active sites is larger than that between the same mimics in combination with the native $[4Fe\text{-}4S]_H$ subcluster (see table 2). As discussed in the main text, the S-to-Se substitution in the $[4Fe\text{-}4S]_H$ cluster reduces the intracluster exchange interactions and, in general, the competing exchange mechanisms (Heisenberg, double exchange, super exchange) attain comparable contributions complicating the magnetic structure of the H-cluster significantly.

Table 2: g-values of HydA1-ADT/-PDT/-PDSe with either the $[4Fe\text{-}4S]_H$ or a $[4Fe\text{-}4Se]_H$ cluster.

HydA1-	$[4Fe\text{-}4S]_H$			$[4Fe\text{-}4Se]_H$		
ADT	2.102	2.040	1.998	2.074	2.020	2.004
PDT	2.095	2.039	2.001	2.094	2.038	1.994
PDSe	2.097	2.052	2.004	2.097	2.055	2.010

6. pH dependence of [4Fe-4Se]$_H$ HydA1-PDT/-PDSe

For [4Fe-4Se]$_H$ HydA1-PDT and -PDSe a proton coupled event at pH 6.0 was observed in FTIR (see fig. S8). Interestingly, only in the reduced state this pH effect can be detected. It results in a blue shift of 1-3 cm^{-1} with respect to the H$_{red}$ state at pH 8.0. Because of the small shift the proton coupled event occurs most likely at the [4Fe-4Se]$_H$ subcluster. Whether this involves a direct protonation of a ligating cysteine or an amino acid proximal to the subcluster, remains unknown. Senger *et al.* analyzed [4Fe-4S]$_H$ HydA1-PDT and suggested a protonation at a coordinating cysteine (C417) of the reduced [4Fe-4S]$_H$ cluster with an overall configuration of: H$^+$[4Fe-4S]$_H^+$ [Fe$_p$(I)Fe$_d$(II)].[7]

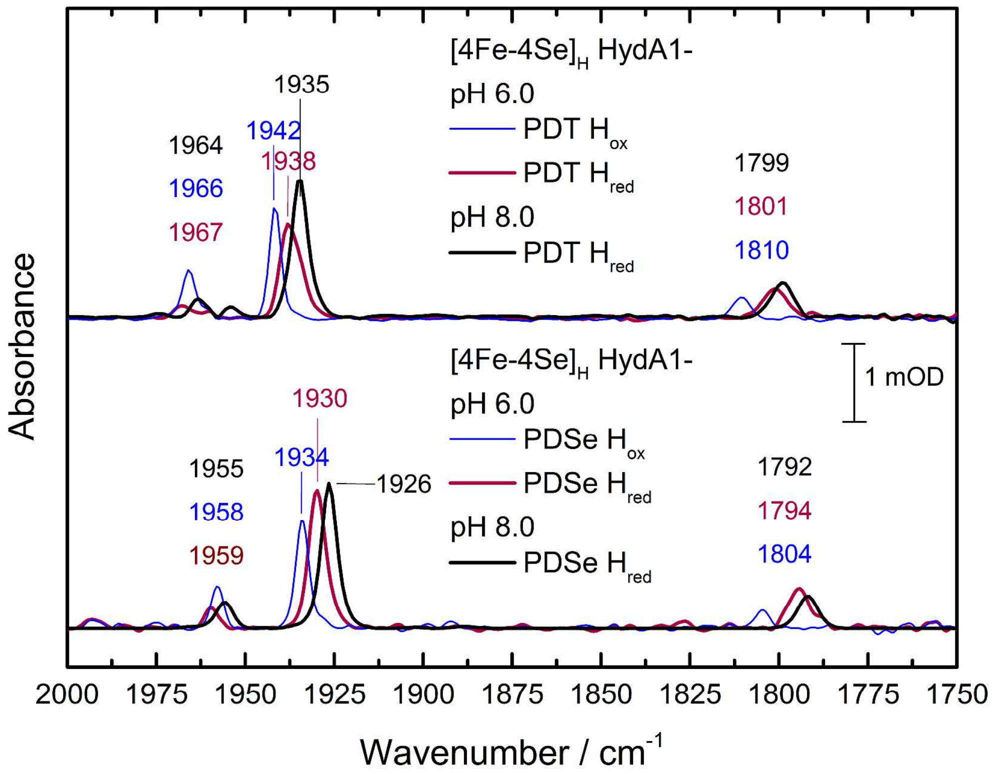

Fig. S8 FTIR spectra of [4Fe-4Se]$_H$ HydA1-PDT and –PDSe in the oxidized (blue) and reduced (red) state at pH 6.0 and pH 8.0. To emphasize the observed blue shift in [4Fe-4Se]$_H$ HydA1-PDT/-PDSe H$_{red}$ the corresponding reduced spectrum at pH 8.0 is superimposed (black). Spectra are taken at 288 K with a resolution of 2 cm^{-1}

Reference List

1. Siebel JF, Adamska-Venkatesh A, Reijerse E, Lubitz W (2015) Biochemistry 54:1474-1483
2. Rumpel S, Ravera E, Sommer C, Reijerse E, Farès C, Luchinat C, Lubitz W (2017) J. Am. Chem. Soc. 140:131-134
3. Silakov A, Wenk B, Reijerse E, Albracht SPJ, Lubitz W (2009) J Biol Inorg Chem 14:301-313
4. Pandey AS, Harris TV, Giles LJ, Peters JW, Szilagyi RK (2007) J Am Chem Soc. 130:4533-4540
5. Gaillard J, Moulis JM, Auric P, Meyer J (1986) Biochemistry 25:464-468
6. Adamska-Venkatesh A, Krawietz D, Siebel JF, Weber K, Happe T, Reijerse E, Lubitz W (2014) J.Am.Chem.Soc. 136:11339-11346
7. Senger M, Laun K, Wittkamp F, Duan J, Haumann M, Happe T, Winkler M, Apfel UP, Stripp ST (2017) Angew. Chem. Int. Ed. 56:16503-16506
